# Can Watching Online Videos Be Addictive? A Qualitative Exploration of Online Video Watching among Chinese Young Adults

**DOI:** 10.3390/ijerph18147247

**Published:** 2021-07-06

**Authors:** Zeyang Yang, Mark D. Griffiths, Zhihao Yan, Wenting Xu

**Affiliations:** 1 Department of Psychology, School of Education, Soochow University, Suzhou 215123, China; 2 International Gaming Research Unit, Psychology Department, Nottingham Trent University, Nottingham NG1 4FQ, UK; mark.griffiths@ntu.ac.uk

**Keywords:** online video watching behaviors, qualitative study, short-form videos, mukbang, online video watching addiction

## Abstract

Watching online videos (including short-form videos) has become the most popular leisure activity in China. However, a few studies have reported the potential negative effects of online video watching behaviors (including the potential for ‘addiction’) among a minority of individuals. The present study investigated online video watching behaviors, motivational factors for watching online videos, and potentially addictive indicators of watching online videos. Semi-structured interviews were conducted among 20 young Chinese adults. Qualitative data were analyzed using thematic analysis. Eight themes were identified comprising: (i) content is key; (ii) types of online video watching; (iii) platform function hooks; (iv) personal interests; (v) watching becoming habitual; (vi) social interaction needs; (vii) reassurance needs; and (viii) addiction-like symptoms. Specific video content (e.g., mukbang, pornography), platform-driven continuous watching, and short-form videos were perceived by some participants as being potentially addictive. Specific features or content on Chinese online video platforms (e.g., ‘Danmu’ scrolling comments) need further investigation. Future studies should explore users’ addictive-like behaviors in relation to specific types of online video content and their social interaction on these platforms.

## 1. Introduction

The number of online video watchers in China reached 927 million by the end of 2020, an increase of 76.33 million since March 2020, and accounting for 93.7% of the total number of Chinese internet users [1]. Among various online applications (e.g., shopping, mobile payment, gaming, etc.), short-form video watching had the highest growth at 12% [1]. The number of online ‘short-form’ video watchers in China reached 873 million at the end of 2020, an increase of 100 million since March 2020, accounting for 88.3% of the total number of Chinese internet users [1].

According to a recent report, 23.9% of the new internet users in 2020 began with online video watching applications [2]. The users of the Chinese online video platforms are mainly aged 20–29 years (89.7%) and highly educated young adults (undergraduates and above; 90.2%) [2]. Watching online videos provides pleasure for billions of individuals worldwide, but excessive use is of potential concern. A representative nationwide survey reported that the prevalence of internet addiction among Chinese college students (*N* = 6929) was approximately 13% [3]. Online video applications might be one type of application contributing to this figure in that they are designed to capture users’ interests, which for some might result in excessive and/or problematic internet use [4]. Online video platform operators use specific statistical analysis approaches and data mining techniques (typically algorithms) to exploit the platform users’ preferences and create bespoke video recommendations more effectively [4]. Therefore, research exploration of online video watching behaviors among Chinese users is warranted.

### 1.1. Generalized Versus Specific Problematic Internet Use

Research dating back 25 years shows that excessive internet use may have some detrimental effects, and that for a minority, it is potentially addictive [5,6]. Since then, many studies have focused on problematic online behavior, including pathological internet use (PIU) [7], internet use disorder (IUD) [8,9], problematic mobile phone use [10], and social networking site addiction [11]. Griffiths argues that any behavior comprising what he considers are the six core components of addiction (i.e., salience, mood modification, tolerance, withdrawal, conflict, and relapse) can be defined as a true behavioral addiction [12].

Davis distinguished two forms of PIU: generalized PIU and specific PIU [7]. Generalized PIU refers to problematic internet use arising from individuals spending all their time online comprising a number of different applications. Specific PIU refers to the problematic use of a specific application or activity, such as online pornography, online gaming or online gambling [7]. More recently, Brand et al. proposed the Interaction of Person-Affect-Cognition Execution (I-PACE) model, which hypothesizes how specific online addictions might be acquired, developed, and maintained [9]. One specific IUD (i.e., internet gaming disorder [IGD]) was included in DSM-5 [13] and (as gaming disorder: predominantly online) in the ICD-11 [14]. In both cases, functional impairment of an individual’s daily life is key to diagnosis [15].

A growing number of studies have focused on specific problematic online activities, such as online gaming disorder [16], online gambling disorder [17], Facebook addiction, [18], Instagram addiction [19], and YouTube Addiction [20], as well other specific IUDs (e.g., online pornography use disorder, online shopping disorder [21]). However, the concepts of generalized PIU and specific PIU remains debatable [22]. For instance, Griffiths has long advocated that there is a fundamental difference between addictions on the internet and addiction to the internet [23,24]. Therefore, in addition to the clarification of the concept of specific PIU, it appears necessary to investigate the problematic or addictive involvement of specific online activities separately. One such example is online video watching.

### 1.2. Problematic Online Video Watching Behaviors

A number of recent studies have investigated problematic video watching-related behaviors, including short-form online video watching addiction [25], problematic YouTube use [20,26,27], problematic online binge watching of television series [28], and problematic mukbang watching [29,30]. These studies all used self-reported psychometric scales to assess problematic online video watching (e.g., short-form video addiction, YouTube addiction, and problematic mukbang watching), typically using scales modified for assessing social media addiction and based on six components of behavioral addiction outlined in the addiction components model [12]. For example, in order to assess YouTube addiction, Balakrishnan and Griffiths adapted the six items of the Bergen Facebook Addiction Scale and replaced the word “Facebook” with “YouTube”. Zhang et al. investigated the Chinese short-form video application TikTok [25]. They found that short-form online video watching addiction was predicted by social anxiety, social isolation, platform personalization, and entertainment, which were mediated by interpersonal and site attachment. De Berail et al. found that parasocial relationships (i.e., the relationship between individuals and others, such as ‘YouTubers’ or celebrity idols that they do not personally know) mediated the association between social anxiety and YouTube addiction [27]. Furthermore, there are some online videos with more specific content, for example, mukbang (‘eating broadcasts’). Kircaburun et al. found positive associations between problematic mukbang watching, eating disorders, and internet addiction [29]. Balakrishnan and Griffiths identified complex relationships between social gratification, content gratification, and YouTube activities and YouTube addiction [20]. Based on these few studies, it appears that problematic/addictive watching of online videos may exist in some contexts, with social interaction, opportunities for personalization, and specific video content being the factors that are most associated with the problematic behavior.

In China, several online video platforms with large numbers of users have emerged, including ones with short-form videos, such as Bilibili and TikTok [31]. TikTok is one of the most popular short-form video applications globally and contains very short 15-s videos. Socially interacting with others was found to predict addiction to this short-form video platform [25]. In addition to TikTok, there are other Chinese short-form video applications (e.g., Kuaishou, Meipai) that require further investigation [25]. Another emerging Chinese video platform is Bilibili, which contains both short-form videos and other more regular videos. Zhang and Cassany investigated the scrolling commenting system on Bilibili (called ‘Danmu’ comments in Chinese), which float across the online video frame while playing [32]. They found that video watchers used repetitive or specific expressions and funny words to interact with each other on Danmu while watching. Danmu comments appear to be an important space for social interaction.

Given that social interaction issues appear to be closely related to problematic online video watching (based on the aforementioned literature), it is important to know whether potentially addictive behaviors exist on the video platforms with various social functions, such as Danmu comments. Research evidence concerning potentially problematic or addictive behaviors on emerging Chinese online video platforms remains limited. Therefore, research exploration on how online video watchers in China behave on these platforms is warranted because Chinese individuals cannot easily access the websites, such as YouTube, from within the country.

### 1.3. The Present Study

Based on the aforementioned studies, there is some research evidence suggesting the existence of online video watching addiction and its potential predictors. However, all of this limited research is quantitative and little of it is in-depth. Therefore, qualitative research is needed to focus more deeply on motivations to watch online videos, how such activity makes individuals feel, and the other potential effects (both positive and negative). Therefore, the present study is qualitative and exploratory, and does not have any hypotheses. Chinese video platforms, such as Bilibili, have specific functions (e.g., Danmu), which are not featured on YouTube. Zhang et al. claimed there were potential demographic and cultural differences across different online video applications, but no details were provided [25]. Therefore, for the specific features and diversity of the Chinese video platforms, the present study does not focus on one specific platform, but explores a wider range of online video watching behaviors. The study aims to investigate online video watching behaviors, related factors (antecedents and consequences), and the potential for addiction among Chinese young adults. Themes relating to the research aims (e.g., watching behaviors, antecedents, and/or consequences) identified from the participants’ interviews are presented qualitatively. Some themes include both antecedents and consequences of online video watching.

## 2. Materials and Methods

### 2.1. Participants

Given that the highest proportion of Chinese online video platform users are 20–29 years old (89.7%) with bachelor’s degree or above (90.2%) [2], the study focused on this age group for recruitment purposes. Twenty young Chinese adults (thirteen females and seven males) volunteered to take part in this study. Participants comprised undergraduates (*n* = 11), postgraduates (*n* = 8), and an office worker (*n* = 1) with bachelor’s degrees, aged 20 to 24 years. The average age was 21.70 years (*SD* = 1.13).

### 2.2. Measures

A semi-structured interview was designed to investigate participants’ online video watching behaviors, antecedents, consequences, and other perceptions. There were five main research questions: (i) which online video platforms do individuals usually use? (ii) What is the focus of the online videos that are watched? (iii) What are the antecedents/motivations of watching online videos? (iv) What are the consequences of online video watching? (v) Are there any addictive signs in online video watching? These questions were proposed based on the research aims of investigating the motivations for online video watching behaviors, related factors (antecedents and consequences), and the potential for addiction among Chinese young adults. All participants were assumed to be normal consumers of online video watchers prior to data collection.

### 2.3. Procedure

Participants were recruited using convenience sampling and snowball sampling. They received invitations—face-to-face invitations in class by the first author and online messages in WeChat groups. Interviews were carried out after they gave their consent to participate. In-depth interviews were conducted, of which seven were online through voice call on WeChat, and 13 were face-to-face. The offline interviews lasted approximately 30 min and were recorded and transcribed.

### 2.4. Ethics

All interviewees gave their informed consent to participate in the study and all participants were assured that their data were anonymous and confidential. The study was approved by the ethics committee of Soochow University (research grant number: 21XM1004). This research was conducted in accordance with the Declaration of Helsinki. All data collection conformed with data protection regulations.

### 2.5. Data Analysis

Thematic analysis was used to analyze the qualitative data from the interviews [33]. The six stages of thematic analysis were followed: (i) data familiarization (reading through all transcripts and getting familiar with the main ideas); (ii) initial coding (conducting coding in several transcripts and identifying initial codes); (iii) searching for themes (initial codes being sorted in broader themes); (iv) reviewing themes (themes being reviewed and refined by reading through all data); (v) defining and naming themes (research team discussing and agreeing on the refined names of the themes); and (vi) producing a report (writing up the results). Computer-assisted qualitative data analysis software (CAQDAS) was used and the interview transcripts were inputted into NVivo Version 11 (QRS International, Melbourne, Australia).

## 3. Results

Eight themes were identified using thematic analysis comprising: (i) content is key; (ii) types of online video watching; (iii) platform functions; (iv) personal interests; (v) watching becoming habitual; (vi) social interaction needs; (vii) reassurance needs; and (viii) addiction-like symptoms. All eight themes can be categorized as comprising the research aims of investigating the motivations for online video watching behaviors, antecedents, consequences, and potentially addictive signs and consequences. Given that this is a qualitative paper, exact numbers are not necessarily important because qualitative research does not try to generalize findings. However, sometimes words are used in this section to give approximate indications of numbers: ‘most’ (more than 15 participants); ‘many’ (10–15 participants); ‘some’ (4–9 participants), and ‘a few’ (less than four participants).

**Theme 1.** Content is key.

The content of the videos was reported as the main focus of online video watching. This theme is part of the research aim of motivations for online video watching behaviors. Most participants reported a range of video content, including mukbang, television series, funny clips, reality shows, travel clips, education, films, gaming, shopping, pornography, etc. One of the increasingly popular types of funny clips was ‘auto-tune remix-themed content’ (‘Gui-Chu’ in Chinese):

*“I think ‘Gui Chu’ is a repetition of an action or a short video clip, and if you watch the video several times, you may think this video is really funny”*.

(Participant 8, female, 20 years)

‘Auto-tune remix-themed content’ videos are mainly found on Bilibili, which contains repetitive audio or videos clips usually with funny film editing. It has been defined as *“a specific genre in which a song is made by completely splitting and re-editing voice sources collected from various audio or video”* [34] (p. 158). Another popular type of funny clips video is the ‘rustic video’ (‘Tu-Wei’ in Chinese), which is mainly presented on short video platforms with ‘corny’ jokes and catchy background music (which can be easily remembered and repeated and may become ‘earworms’), and usually filmed in the countryside. For example, Participant 3 reported that her roommate always played ‘rustic videos’ in which the individuals had a *“pretentious performance with weird makeup”.* Participant 5 appeared to dislike this type of online video:

*“My roommate often watches ‘Tu Wei’ videos for a long time, but these videos are not attractive to me. I think the background music of these videos is annoying and sometimes brain washing [catchy], and the content is also vulgar and even unnatural”*.

(Participant 5, female, 22 years)

In addition to the content of the video itself, the content of Danmu (scrolling bullet comments) and regular comments were mentioned as an important focus during online video watching by 15 participants. A few participants (3 out of 15) said they loved to see Danmu and comments while watching videos. For example:

*“I must watch Danmu while watching videos. I think Danmu is extraordinarily interesting. It is less funny when you watch video without Danmu”*.

(Participant 16, female, 22 years)

*“There is no soul without comment. Sometimes it is the comments rather than the content itself that makes a video interesting”*.

(Participant 18, female, 23 years)

However, some participants (6 out of 15) reported that they preferred to watch videos without Danmu unless they need to see the others’ ideas at some point in the video. This appears to indicate the social purpose of viewing Danmu comments.

*“I usually turn off Danmu while watching video unless I want to know what the others are thinking…I get rid of Danmu because it affects my watching experience”*.

(Participant 11, female, 21 years)

Overall, online video watchers not only pay attention to the content of the video, but also to the content of Danmu or comments presented together with the video. This shows the social function of online video watching (also discussed further below).

**Theme 2.** Types of online video watching.

Online videos were differentiated by the participants as being short or regular, and livestream or recorded. This theme also concerns the motivations for online video watching behaviors. Some described short videos as being less than one minute. They preferred to watch short videos because they could watch them in their spare time.

*“I prefer short videos to regular videos. Short videos are often less than one minute, sometime about ten seconds, which meets my needs of watching videos in small time windows. And short videos are often more entertaining than other types of videos”*.

(Participant 12, female, 21 years)

One participant explained that short videos were becoming more popular because the pace of life is speeding up.

*“I think the rise of short video is inevitable. Nowadays, the pace of society is getting faster and faster. People are more willing to spend fragmented time on short videos. For example, I’m following an online news channel on Bilibili. Their videos often last for only several minutes, which saves my time a lot”*.

(Participant 20, male, 21)

Most participants talked about recorded online videos only, although 7 out of 20 noted that they watched livestream selling (i.e., online live shows for marketing or promotion, with simultaneous product ordering) (Participants 9, 16, 17), live gaming (Participant 14), and personal livestream shows (leisure talk or singing performances hosted by influencers or famous live-streamers) (Participants 4, 11, 12).

**Theme 3.** Platform function hooks.

Online video watching behaviors were greatly affected by the functional and technological hooks of online video platforms, including push notifications, recommendations, and auto scrolling. This theme concerns the research aim for the antecedents for online video watching. Participants (9 out of 20) were clearly aware of data mining technology when they received push notifications or recommendations accurately adapting to their interests.

*“I am aware that the recommendations from the video platforms are based on algorithms. For example, when I watched several mukbang videos, the platform recommended more mukbang videos to me until I intentionally changed to another video type”*.

(Participant 2, male, 22 years)

*“If I haven’t used this app today, it may then push some notifications to me that attracts me to use it. And the apps often recommend something similar to what I searched or said before. Now many apps have these functions”*.

(Participant 17, female, 22)

Another important function was the auto-scrolling function, which allows continuous watching by automatically skipping to the next video.

*“After I finished a video clip, the platform will play the next video automatically, and then I may find that video is also interesting and continue to watch videos”*.

(Participant 16, female, 22 years)

**Theme 4.** Personal interest.

All 20 participants reported that the main reason for watching online videos was their personal interest and/or their need for information. This theme concerns the antecedents and motivations for online video watching. They watched videos because they were interested in specific content or topics as noted above. Some participants wanted to seek help from the internet for specific skills, learning sources, and gaming skills. Some reported that they watched videos because of ‘idol worship’. For example:

*“I watch online videos mainly because of my favorite female pop group”*.

(Participant 13, male, 23 years)

Once they acquired knowledge and skills, or specific information (e.g., mukbang, idol’s reality show), they received content gratification. This gratification motivated further watching behaviors. For example:

*“I usually watch mukbang when doing exercise. When watching the others eating on camera, I feel as if I am also eating the delicious food. That makes me psychologically gratified and motivates me to watch more mukbang videos”*.

(Participant 10, female, 20 years)

**Theme 5:** Watching becoming habitual.

Watching online videos can be motivated by existing habits, but, at the same time, one could develop new habits. This theme concerns the motivations, antecedents, and consequences of online video watching. Three participants’ narratives suggest that online video watching behaviors can become life habits, similar to watching television in their childhood. This was not seen as harmful to their lives.

*“When I was a child, I always watched cartoons after dinner every day at around 5 pm. Now I watch online videos instead. I usually check if some of my following uploaders have updated their channels at 7 or 8 pm. It is more like a habit, and I don’t think it is harmful to my life”*.

(Participant 20, male, 21 years)

However, only participant 20 compared online video watching with cartoon watching experiences from his childhood. It does not appear that different individuals might have different emotional feelings on typical television watching and online videos.

One participant said she developed a habit of watching online mukbang videos during gym exercise:

*“I think watching mukbang videos on Bilibili has become a habit, which meets my needs while exercising”*.

(Participant 10, female, 20 years)

Participant 13 noted that watching or listening to livestream leisure talk or chatting with fans hosted by favorite idols was a daily practice.

*“Watching live stream, usually chatting with fans, by my idol has become a daily activity for me. Sometimes I just put my phone down and listen to it while doing the other things”*.

(Participant 13, male, 23 years)

One participant believed that her online video watching was a habit rather than an addiction:

*“I have a habit of watching mukbang, but I am not addicted to it. I usually watch four or five mukbang videos each day, and it won’t take more than half an hour”*.

(Participant 10, female, 20 years)

**Theme 6:** Social interaction needs.

Some participants mentioned social interaction issues when talking about both the antecedents and consequences of online video watching. First, many participants watched the videos because of their social needs (e.g., fear of missing out (two participants), peer influences (five participants), and parasocial relationship with uploaders (five participants)). Similar to other social media, online video watching was motivated by social needs, especially the need for peer interaction, and to be able to speak to friends about the same things they had watched.

*“Some of my friends may talk about a popular video or a TV show. If I haven’t watched them yet, I can’t understand their topics or join them. That made me frustrated”*.

(Participant 1, male, 22)

Five online video watchers established their own social codes or signals that are described as ‘memes’ (“Geng” in Chinese). Only when all the interlocutors know the memes can they jointly understand a joke. This again showed the social nature of watching online videos. For example:

*“The memes are the ways of communication with the others. Many people around you are talking about the memes. If you know the memes, you will know what they are talking about. It is a necessity for social interaction”*.

(Participant 12, female, 21)

Some participants (5 out of 20) established parasocial relationships with the video uploaders they were following. They felt a need to constantly check the uploaders’ updates, just like keeping in touch with friends. For example:

*“I frequently check if some of my following uploaders have updated new videos. If not, I may feel disappointed and lost”*.

(Participant 2, male, 22 years)

*“Some video uploaders or live stream hosts have become my virtual friends. Just like my old friends, I will check their lives when I think of them”*.

(Participant 12, female, 21)

Moreover, watching online videos can have positive and negative social impacts. Positively, some participants (7 out of 20) gained social rewards because they felt pleased and gratified in social interaction during watching.

*“I think watching live stream is interactive and immersive. It feels like you are communicating with the live stream host closely, and the host is talking to you. Although I know it is not real, I get much gratification. I often watch live stream at midnight”*.

(Participant 12, female, 21 years)

*“Some video platforms have their own ‘Danmu culture’. Thousands of people chat on Danmu. It makes you feel like that you are watching the recorded video together with a lot of people simultaneously. Danmu definitely makes the video more enjoyable”*.

(Participant 2, male, 22 years)

Negative impacts included social conflicts with family, friends, and strangers in public when watching online videos was too much or too loud.

*“One of my roommates often plays videos too loudly, which makes me uncomfortable and even disgusted”*.

(Participant 3, female, 22)

Sometimes, social conflicts and even cyberbullying occurred in comments and Danmu when online video watchers disagreed with each other. Participants 3, 11, and 20 all described some users as “*internet trolls*” who always initiated social conflicts. Participant 2 witnessed that some users quarreled on Danmu. Participant 10 reported her experience of cyberbullying:

*“Once I commented on a video, a stranger refuted me with ten more comments…There must be something wrong with him!”*.

(Participant 10, female, 20 years)

**Theme 7:** Reassurance needs.

This theme, like that of social interaction, includes both antecedents and consequences of online video watching. For some participants, watching online videos was motivated by reassurance needs and could cause mood changes. Some participants (7 out of 20) watched online videos because of their reassurance need for recreation and escapism. For example, Participant 10 watched mukbang videos and said:

*“I usually do gym exercise in the evening. It is always a little bit boring and I’m often hungry so I watch mukbang videos. Watching the others eating makes me feel like I have taken their food too, which provides me with reassurance and satisfaction”*.

(Participant 10, female, 20 years)

Participant 2 tried to escape from tasks at hand by watching online videos:

*“Watching online videos makes me procrastinate more. Sometimes I plan to finish my tasks after watching the video, but I always put it off to the next day. I may escape from my tasks by watching videos”*.

(Participant 2, male, 22 years)

Second, there were positive or negative mood changes that were facilitated by online video watching. Some participants reported feelings of relaxation, encouragement, and being virtually accompanied after watching the online videos. For example:

*“Watching online videos is an effective way to positively change my mood especially when encountering difficulties or disappointments”*.

(Participant 13, male, 23)

*“Many uploaders share daily lives with their pets. My cat is not with me and I always miss it. Watching these videos makes me feel reassured and compensated”*.

(Participant 12, female, 21 years)

*“If the online video is not played, the task at hand might be paused or stopped, [The video] gives me a feeling of being accompanied by others”*.

(Participant 9, female, 20 years)

However, there were negative mood changes, such as when their favorite gaming team lost:

*“When my supported gaming team lost, I felt disappointed and frustrated in the following two or three days. That’s the negative emotional impact of the videos”*.

(Participant 4, male, 22 years)

Participant 7 watched videos because of boredom, but gained more boredom and feelings of hollowness after watching. She appeared to be trapped in a vicious circle:

*“I usually watch online videos when I’m bored. But after watching for a while, I may feel even more bored and empty”*.

(Participant 7, female, 24 years)

**Theme 8:** Addiction-like symptoms.

Addiction or addiction-like symptoms were mentioned by 18 participants in relation to specific video content (e.g., pornography, mukbang, television series, live gaming), short videos (e.g., continuous scrolling), platform push notifications, social conflicts, time wasting, and physical issues. This theme can be categorized as concerning the potential of addictive watching. Specific content, pornography for example, was described as addictive:

*“One of my friends is addicted to online pornography. She watches porn almost every day, sometimes even excessively. Then she always feels exhausted and easily distracted because of watching too much porn”*.

(Participant 5, female, 22 years)

Television series, movie series, reality shows, and uploaders’ video series were perceived as addictive because some participants watched the episodes continuously and uncontrollably. Since the complete content was split into different clips, Participant 4 stayed up to finish the series.

*“Sometimes I stay up late to watch online series, I just can’t stop it. Because the series often have suspense at the end of one episode, it motivates me to watch the following episode continuously. That leads to poorer sleep quality and makes me tired all day”*.

(Participant 4, male, 22)

As discussed previously, platform push notifications were the key reason for continuous online video watching:

*“You can always receive push [notifications] and you enjoy watching the recommended videos. The platform knows your interests and favorites”*.

(Participant 1, male, 22 years)

*“The platform’s push [notifications] related video comes to you automatically and then you would find this video also interesting. I used to watch short videos all day until I ran out my phone’s power. That was horrible! I uninstalled that short video app”*.

(Participant 16, female, 22 years)

In addition to content, one participant also highlighted the convenience of watching continuously, which is brought by specific functions of short-form videos:

*“With my phone in hand, I feel too easy for me to scroll down to the next video. It won’t be that easy when I rotate my phone 90 degrees and watch videos horizontally”*.

(Participant 16, female, 22 years)

Time wasting was frequently reported by some as a negative consequence of online video watching. Participants admitted they wasted too much time (*“longer than expected”*, Participant 2) especially watching short online videos. Addiction-like symptoms, such as regrets and social conflicts, were also mentioned by some. For example:

*“Sometimes I keep scrolling on the video app for a whole afternoon, It’s horrible. Before an important examination, I was anxious but still watched short videos for a long time. I felt serious regret, but I just could not stop watching videos. That also raised some conflicts, such as quarreling with my parents”*.

(Participant 7, female, 24 years)

Physical issues were also reported by a few participants as a negative effect of online video watching:

*“Watching videos excessively has bad effects on my vision, and sometimes my neck hurts”*.

(Participant 7, female, 24)

## 4. Discussion

The present study identified eight themes in relation to online video watching: (i) content is key; (ii) types of online video watching; (iii) platform function hooks; (iv) personal interests; (v) watching becoming habitual; (vi) social interaction needs; (vii) reassurance needs; and (viii) addiction-like symptoms. In relation to the research questions: (i) online video watchers’ key focus was on both the video content and the associated comments (including simultaneous Danmu comment); (ii) the antecedents for watching online videos included platform hooks, such as push notifications, personal interests, established watching habits, social interaction needs, and reassurance needs; (iii) the consequences of watching online videos included habit development, social rewards or effects, and positive or negative reassurance; and (iv) addiction-like symptoms were self-reported by some participants.

Participants watched online videos for social interaction, educational content, and relaxation and it was the positive effects on online video watching that were most mentioned. Only very specific online video content was perceived as addictive (e.g., pornography, mukbang, boxed television series, etc.). Additionally, short-form videos (especially TikTok videos on smartphones) were reported as addictive and time wasting due to continuous scrolling functions on smartphones. Push notifications from the online video platforms were reported as the key drivers for potential addictive watching. Physical issues were also mentioned by a few participants as addiction-like symptoms. However, participants’ perceived ‘addiction-like’ symptoms in the present study do not indicate addiction or disorder as this cannot be confirmed unless there is a clinical diagnosis and clear evidence of daily functional impairment (of which there was little).

In line with previous theoretical models and empirical studies, the present study’s findings concur that social interaction plays an important contributory role in online video watching [18,20,25]. Participants noted that they established a kind of virtual relationship with video uploaders and other watchers. Participant 2 said he felt “disappointed” when there was no update from his following uploaders. This suggests a craving or desire for new video updates, which was one phase in the gratification-compensation process described in Brand et al.’s I-PACE model [9]. Brand et al. noted that cue-reactivity and craving can be associated with addiction [9]. Participant 2 reported addiction-like symptoms and consequences, including poor sleep quality, being unable to stop watching online videos, and time wasting, together with his strong craving for uploaders’ updates. This appears to confirm previous findings that engaging in relationships with YouTubers was a predictor of YouTube addiction [27]. However, whether this participant was truly addicted to online videos cannot be made on the basis of the interview alone. Furthermore, an earlier study reported that information-seeking and entertainment were the most important motives for internet use, while interpersonal communication only played a limited role [35]. Consequently, further quantitative studies to investigate the role of social interaction on internet use or problematic internet use are warranted.

Similar to previous studies ([20,29,30]), the study’s findings confirm the importance of specific online content in relation to potential addiction. Participants tended to refer to specific video content (e.g., pornography, mukbang) when talking about addiction-like symptoms. Therefore, in addition to discussing addictions to specific platforms (e.g., YouTube addiction), research attention also needs to be paid to potential addictions to specific content, such as mukbang addiction. It is possible that these specific video watching addictions might just be the reflections of the other behavioral addiction or disorders such as eating disorder. For example, disordered eating was found to be positively associated with problematic mukbang watching behaviors in a previous study [29].

Besides addictions to specific content, concepts, such as short-form video addiction [25], remains unclear and debatable. Short-form videos need to be clearly defined, as they were defined as 15-s videos in one previous study [25], whereas short-form videos in the present study were described as videos less than one minute in length. Short-form video watching was reported as addictive or problematic because of the ‘prepared’ video content (push notifications based previous viewing behavior) and the auto-scrolling function. Participants gained reinforced gratification while repeatedly skipping through the continuous clips, which could be explained by the recent theoretical model of specific IUD (e.g., I-PACE model [9]). Addictive or problematic short-form video watching behaviors might be similar to the other online behavioral addictions (e.g., specific social media addictions).

In line with findings in the extant literature [36,37], the study identified self-perceived negative psychological effects of online activities among a small minority of individuals (in this case, online video watching). Some participants reported that negative mood might be elicited by social conflicts in the simultaneous Danmu comments while watching or other post-video comments. This socially-related negative effect can also play a contributory role in other behavioral addictions/disorders, for example, social networking site addiction [38]. However, the relationship between online video watching behaviors and negative psychological effects is complex because the virtual social context needs to be considered alongside the video content. Social conflicts appear to be one specific bridge between online video watching behaviors and negative mood states, and warrants further research. A recent systematic review reported that excessive social media use had limited impact on people’s well-being [39]. This might also be explained by the bridging factors, such as social conflict, between social media use and well-being.

Similar to a qualitative study on TikTok [40], the present study found that individuals watch short video videos in order to keep a “*fashionable lifestyle*” or keep up-to-date with peers. This has also been reported in previous qualitative studies on problematic smartphone use where individuals reported the importance of social pressure and peer pressure in their continued smartphone use [37]. Moreover, similar to previous studies [40], the catchy background music in TikTok was mentioned in the present study. However, participants in Lu and Lu’ s study reported positive feelings concerning the catchy music while participants in the present study said they were annoyed by the catchy music. Consequently, the effect of such background music on subsequent behavior in short video watching needs further exploration.

The present study identified a number of types of online video watching that have not been sufficiently investigated in previous studies, including: videos with Danmu comments (i.e., comments floating across the screen while the online video is playing), videos with auto-tune remix-themed content (i.e., videos containing repetitive audio or videos clips usually with funny film editing), and rustic videos (videos with corny jokes usually filmed in the countryside). Danmu comments have previously been studied from a linguistics perspective [32], but were not associated with potential addictive or problematic internet use. Participants in the present study expressed diverse behaviors towards Danmu. For instance, some shut down Danmu comments while others focused on Danmu constantly when watching the online videos. Social conflicts, social gratification, and sense of parasocial relationships were reported in relation to Danmu comments. Considering the close relationship between problematic video watching and social interaction noted in previous studies, further investigation as to whether Danmu comments are associated with online video watching addiction is needed. Furthermore, the auto-tune remix-themed content and rustic content in Chinese online video platforms were reported as either attractive or annoying among different individuals. Therefore, further investigation is required into these specific forms of emerging video content in relation to habitual behavior.

There are some limitations in the present study. First, the participants might have given socially desirable answers in the interviews. They might have exaggerated or concealed some behaviors or feelings concerning their online video watching. This issue needs to be addressed, even though the interviewer in the present study was experienced in conducting interviews for qualitative research. Second, surveys and psychometric instruments were not used in the present study. Future studies could combine different data collection methods to triangulate with qualitative data. For example, the items assessing YouTube addiction used by Balakrishnan and Griffiths [20] could be adapted to assess potential addictive behaviors among Chinese video platform watchers. Other methods, such as focus groups and participatory action research, could be used to collect data on this topic. There are clearly other methods (additional to one-to-one interviews) that could be employed to investigate the environmental and individual effects concerning online video watching usage. Another potential limitation might be the convenience sample because their responses only represent the perceptions of a very small number of participants. Future studies might recruit a larger sample with different age levels using stratified sampling to further explore online video watching. Other future studies should attempt to use clinical samples of treatment-seeking individuals as none of the individuals in the present study were known problematic users.

There are also some possible directions for future studies. First, more studies should focus on addictive or problematic watching behaviors relating to specific video content (e.g., auto-tune remix-themed content). Second, future studies need to investigate whether short-form video addiction exists as a single entity or whether it simply belongs to the more generic category of social media addiction. Third, Danmu comments, as a typical function of Chinese video platforms, should be explored from other perspectives, including linguistics and communication. Fourth, future studies might use (as aforementioned) other data collection methods (e.g., focus groups and social network analysis) to investigate problematic online video watching behaviors as a consumption problem from a social perspective. Future studies need to control and select the samples’ characteristics carefully, in order to corroborate relevant research evidence. It should also be noted that the present study mainly focused on the watching behaviors on video platforms, whereas the uploading of video content was not included. Since online video platforms, such as YouTube, Bilibili, and TikTok have both video uploading and sharing functions [20,31], it is therefore necessary for future studies to investigate online video consumption more generally, including video watching, uploading, and sharing behaviors. The frequency of video applications usage and the total time spent on video watching are the possible directions for data collection. Finally, further research should also investigate whether individuality and the capacity for abstraction when individuals enter the microworld of their smartphone has the same emotional, familial, and relational impact as the habit of watching cartoons or similar television programs.

## 5. Conclusions

The present study explored online video watching among Chinese young adults, using a qualitative design. In line with previous studies, in addition to information seeking and entertainment, social interaction was a key driver in online video watching. Furthermore, addiction-like symptoms were reported regarding specific video content (e.g., pornography), program types (e.g., short-form video, series box-sets), and continuous watching driven by platform push notifications. This study contributes to the limited literature on problematic video watching behaviors on Chinese emerging platforms (e.g., Bilibili, TikTok), which have different features from the other widely used online video platforms, such as YouTube. Further investigations are needed on problematic watching behaviors of specific online video content (e.g., auto-tune remix-themed content), new forms of online social interaction during video watching (e.g., Danmu comments), problematic short-form video watching (e.g., problematic TikTok use), and video sharing behaviors. Different research methods (e.g., mixed-methods designs, and large-scale surveys using stratified sampling with a wider and more representative range of age groups) can be utilized to further explore this topic. Moreover, individual differences and sociodemographic backgrounds need to be considered in future studies concerning online video watching as well as a more detailed analysis of the techniques used by streaming platforms that facilitate repeated viewing (e.g., use of algorithms).

## Data Availability

The data presented in this study are available on request from the corresponding author. The data are not publicly available due to privacy.
